# Artificial intelligence in cardiology: an updated systematic review with ethical considerations and challenges in implementing artificial intelligence models

**DOI:** 10.1097/MS9.0000000000004607

**Published:** 2025-12-19

**Authors:** Dev Patel, Reshmitha Kantamneni, Jabez David John, Tirath Patel, Arnesh Shukla, Ayesha Salma, Nikhilesh Anand

**Affiliations:** a Department of Medicine, Lokmanya Tilak Municipal Medical College, Mumbai, Maharashtra, India; b Department of Medicine, Rangaraya Medical College, Kakinada, Andhra Pradesh, India; c Department of Medicine, Malla Reddy Institute of Medical Science, Hyderabad, India; d Department of Medicine, Trinity Medical Sciences University School of Medicine, Kingstown, Saint Vincent and the Grenadines; e Department of Medicine, St Martinus University, Willemstad, Curaçao; f Department of Medicine, Shadan institute of medical sciences, Hyderabad, Telangana, India; g Department of Medical Education, University of Texas Rio Grande Valley, Edinburg, TX, USA

**Keywords:** Artificial intelligence, Cardiovascular disease, Diagnostic accuracy, Ethical considerations, Patient outcomes

## Abstract

The integration of artificial intelligence (AI) in cardiovascular medicine presents a transformative opportunity to enhance diagnostic accuracy and improve patient outcomes. This systematic review evaluates the impact of AI on cardiovascular diagnostics with a focus on its ability to surpass traditional methods in accuracy, efficiency, and predictive capabilities. Machine learning and deep learning have demonstrated significant advancements in areas such as echocardiography, electrocardiography, computed tomography angiography, and predictive analytics.

AI algorithms have demonstrated superior performance in identifying subtle patterns and anomalies. Additionally, AI has shown promise in predictive analytics, forecasting disease progression and tailoring treatment plans, thereby improving patient outcomes. Despite these advancements, significant gaps remain in our understanding of AI’s full impact on cardiovascular medicine. Challenges such as the generalizability of AI models, ethical considerations, data privacy, issues related to data quality, clear guidelines on AI implementation in clinical practice and potential biases in AI algorithms warrant further investigation.

Key findings indicate that AI systems consistently achieve higher diagnostic accuracy, reduce inter-observer variability, and facilitate earlier detection of cardiovascular conditions, leading to improved patient outcomes. In conclusion, AI holds substantial promise for improving diagnostic accuracy and patient outcomes in cardiovascular medicine. This review provides valuable insights into the benefits and limitations of AI, guiding future research and clinical practice to ensure responsible and effective integration of AI technologies in cardiovascular health care.

## Introduction

The advent of artificial intelligence (AI) has ushered in a new era of technological advancements across various fields, including medicine. In cardiovascular medicine, AI has emerged as a transformative force, promising to revolutionize diagnostic accuracy and improve patient outcomes. This systematic review aims to assess the impact of AI in enhancing diagnostic precision and patient care within the realm of cardiovascular medicine. The impetus for this research stems from the growing body of evidence suggesting that AI can significantly augment traditional diagnostic methods, thereby offering novel solutions to long-standing challenges in cardiovascular health care^[1]^.

**HIGHLIGHTS**

- Artificial Intelligence (AI) Advances in Cardiology: Machine learning and deep learning surpass conventional techniques in fields like echocardiography, electrocardiography, and computed tomgraphy angiography to greatly improve diagnosis accuracy in cardiovascular medicine.
- Patient Outcomes: AI integration improves patient outcomes leading to lower mortality and morbidity via enabling early diagnosis and individualized treatment regimens.
- Challenges: Integrating AI into clinical workflows is challenging due to inconsistent data quality and the need to navigate complex regulatory requirements that can slow down implementation.
- Ethical Considerations: The review emphasizes the critical importance of addressing ethical concerns like bias reduction, algorithmic transparency, and data privacy.
- Future Directions: The appropriate integration of AI in cardiovascular health care will require ongoing study and the creation of comprehensive guidelines.

### Current state of knowledge

Cardiovascular diseases remain the leading cause of mortality worldwide, responsible for an estimated 17.9 million deaths annually. The global burden of these diseases underscores the critical need for timely, accurate, and efficient diagnostic strategies. The current landscape of cardiovascular diagnostics relies heavily on a combination of clinical assessment, imaging techniques, and biomarker evaluation. Traditional modalities such as electrocardiography (ECG), echocardiography, chest radiography, stress testing, and coronary angiography are routinely used in clinical practice. In addition, laboratory testing for biomarkers like troponins, B-type natriuretic peptide (BNP), and C-reactive protein (CRP) plays a key role in evaluating myocardial injury, heart failure, and systemic inflammation.

Despite their proven clinical value, these diagnostic tools are not without limitations. Many are operator dependent and require specialized training for accurate interpretation. For instance, echocardiography, while non-invasive and widely available, can be limited by poor image quality or patient body habitus. ECGs can miss subtle or transient abnormalities, and their interpretation is often subjective, with inter-observer variability. Invasive procedures such as coronary angiography, though highly informative, carry risks and are not suitable for all patients, particularly in low-resource settings or for routine screening purposes.

Moreover, the increasing complexity and volume of patient data present new challenges. Clinicians must integrate findings from multiple sources – imaging, clinical history, labs, and sometimes genetic testing – often under time constraints. This fragmented approach can lead to delayed diagnoses, missed opportunities for early intervention, and suboptimal patient outcomes^[1]^.

Recent technological advancements, particularly in imaging modalities such as cardiac magnetic resonance imaging (MRI), CT angiography, and nuclear imaging, have improved diagnostic capabilities. These techniques offer high-resolution anatomical and functional insights but are resource-intensive, expensive, and often limited to tertiary care centers. Similarly, wearable devices and mobile health technologies are becoming increasingly popular for real-time monitoring of cardiac rhythm and physiological parameters, yet they generate vast amounts of data that require efficient analysis and interpretation.

Given these complexities, there is a growing interest in leveraging artificial intelligence (AI) to enhance diagnostic accuracy, efficiency, and accessibility. AI technologies, including machine learning (ML) and deep learning (DL), have demonstrated the potential to analyze large datasets, identify hidden patterns, and support clinical decision-making with greater speed and consistency than traditional methods. This evolving paradigm aims not to replace clinicians but to augment their capabilities, offering a more integrated, predictive, and personalized approach to cardiovascular care.

ML and DL have shown considerable promise in addressing these limitations. AI algorithms have demonstrated superior performance in image analysis, predictive analytics, and personalized medicine, outperforming conventional methods in several studies. For instance, AI applications in echocardiography, ECG, and cardiac imaging have shown enhanced diagnostic accuracy. AI algorithms can analyze vast amounts of imaging data rapidly and with high precision, identifying subtle patterns and anomalies that may be overlooked by human eyes. Additionally, predictive models using AI have been developed to forecast disease progression and patient outcomes, providing clinicians with valuable insights for early intervention and tailored treatment plans^[2]^.

### Gaps in knowledge

Despite the promising developments, several gaps remain in the current understanding of AI’s impact on cardiovascular medicine. There is a lack of comprehensive guidelines and research outcomes that consolidate the evidence on AI’s efficacy across various diagnostic modalities and clinical settings. Furthermore, the long-term effects of AI integration on patient outcomes and health care systems are not well understood. Issues related to the generalizability of AI models, ethical considerations, data privacy, and the potential for AI-induced biases also warrant thorough investigation^[3]^.

### Research plan and objectives

This systematic review aims to bridge these knowledge gaps by critically evaluating the existing literature on AI applications in cardiovascular diagnostics and patient care. The primary objectives of this review are:

1. To Review: Systematically analyze studies that have implemented AI in cardiovascular diagnostics to determine the extent of its impact on diagnostic accuracy and patient outcomes.
2. To Explore: Investigate the various AI technologies utilized in cardiovascular medicine, their methodologies, and their respective outcomes.
3. To Find Out: Identify the benefits and potential drawbacks of AI integration in clinical practice, including its effect on patient outcomes, workflow efficiency, and health care costs.

### Research questions

The central research questions guiding this review are:

How does AI enhance diagnostic accuracy in cardiovascular medicine compared to traditional methods?

What are the clinical outcomes associated with AI-assisted diagnostics in cardiovascular care?

What ethical challenges and limitations exist in the application of AI in cardiovascular medicine?

### The problem

The primary problem addressed by this research is the need for more accurate, efficient, and reliable diagnostic tools in cardiovascular medicine. Misdiagnosis or delayed diagnosis of cardiovascular conditions can lead to adverse patient outcomes, increased health care costs, and higher mortality rates. By systematically reviewing the impact of AI, this research seeks to provide evidence-based insights and statistical proof that can inform clinical practice and policy-making^[4]^.

### Potential benefits and consequences

If the problem of diagnostic inaccuracy in cardiovascular medicine is effectively addressed through AI, the potential benefits are substantial. Improved diagnostic accuracy can lead to earlier detection of cardiovascular conditions, more precise treatment plans, and better patient outcomes. Health care systems could experience increased efficiency, reduced costs, and enhanced patient satisfaction. Conversely, failing to harness the potential of AI in this field could perpetuate the existing challenges, leading to continued diagnostic errors, suboptimal patient care, and unnecessary health care expenditures^[5]^.

### Importance and objectives

This review is crucial for several reasons. First, it synthesizes the growing body of evidence on AI in cardiovascular medicine, providing a consolidated resource for clinicians, researchers, and policymakers. Second, it identifies best practices and areas needing further research, guiding future investigations and innovations. Lastly, it addresses ethical and practical considerations, ensuring that the integration of AI into clinical practice is both effective and responsible^[6]^.

In summary, the intention of this paper is to provide a comprehensive assessment of the role of AI in enhancing diagnostic accuracy and patient outcomes in cardiovascular medicine. By reviewing, exploring, and finding out the impacts and implications of AI, this research aims to offer valuable insights that can drive advancements in cardiovascular health care.

This manuscript is made compliant with the TITAN checklist to ensure transparency in the reporting of AI^[7]^.

## Methodology

### Research design

This systematic review will follow the Preferred Reporting Items for Systematic Reviews and Meta-Analyses (PRISMA) guidelines. The methodology will encompass a structured process of literature search, study selection, data extraction, quality assessment, and data synthesis.

### Literature search strategy

A comprehensive literature search was conducted across several electronic databases, including PubMed, MEDLINE, Cochrane Library and Google Scholar in June 2025. The search strategy used a combination of Medical Subject Headings (MeSH) terms and free-text keywords relevant to AI applications in cardiovascular diagnostics and patient outcome.

The following search terms were employed:

- Artificial intelligence
- Machine learning
- Deep learning
- Cardiovascular diagnosis
- Diagnostic accuracy
- Predictive analytics
- Patient outcomes

Boolean operators were used to enhance the precision of the search, for example:

“Artificial intelligence” AND “Cardiovascular diseases” OR “Heart diseases”

“Machine learning” AND “Diagnostic accuracy” AND “Patient outcomes” AND “Cardiovascular diseases”

Table 1 shows the databases searched, the search terms used, and the total number of records retrieved from each source.

**Table 1:** Database search strategy and results

Database	Keywords	Search strategy	Results
PubMed	Artificial intelligence, Machine learning, Deep learning, Cardiovascular diagnosis, Diagnostic accuracy, Predictive analytics, Patient outcomes	“Artificial intelligence” AND “Cardiovascular diseases” OR “Heart diseases” AND “Machine learning” AND “Diagnostic accuracy” AND “Patient outcomes” AND “Cardiovascular diseases”	2120
Google Scholar	Artificial intelligence, Cardiovascular disease	“Artificial intelligence” AND “Cardiovascular diseases” OR “Heart diseases”	621
Cochrane	Artificial intelligence, Cardiovascular diagnosis, Diagnostic accuracy, Patient outcomes	“Artificial Intelligence” AND “Diagnostic accuracy” AND “Patient outcomes” AND “Cardiovascular diseases”	442
MEDLINE	Artificial intelligence, Machine learning, Deep learning, Cardiovascular diagnosis, Diagnostic accuracy, Predictive analytics, Patient outcomes	“Artificial intelligence” AND “Cardiovascular diseases” OR “Heart diseases” AND “Machine learning” AND “Diagnostic accuracy” AND “Patient outcomes” AND “Cardiovascular diseases”	274

To ensure the breadth of the search, we used truncation and wildcard operators (AI, cardiovascular) where appropriate. References from key articles were manually searched for additional relevant studies not captured through database searches.

### Study selection criteria

Inclusion and exclusion criteria will be established to select studies relevant to the research question.

#### Inclusion criteria include:

1. Population: Studies involving patients with cardiovascular conditions.
2. Intervention: Application of AI technologies (e.g., ML, DL) in cardiovascular diagnostics.
3. Comparison: Comparison with traditional diagnostic methods or no intervention.
4. Outcomes: Studies reporting on diagnostic accuracy (e.g., sensitivity, specificity, predictive values) and patient outcomes (e.g., morbidity, mortality, treatment efficacy).
5. Study Design: Randomized controlled trials (RCTs), cohorts, comparative studies, and grey literature.
6. Publication Language: Studies published in English.

#### Exclusion criteria include:

1. Studies not involving AI in cardiovascular diagnostics.

2. Studies not reporting on relevant outcomes.

3. Non-English language publications.

4. Grey literature and clinical trials

### Data extraction

Data will be extracted from the selected studies using a standardized data extraction table. Two independent reviewers will perform data extraction to ensure accuracy and consistency. Discrepancies will be resolved through discussion or consultation with a third reviewer.

### Study selection

**Figure 1.**
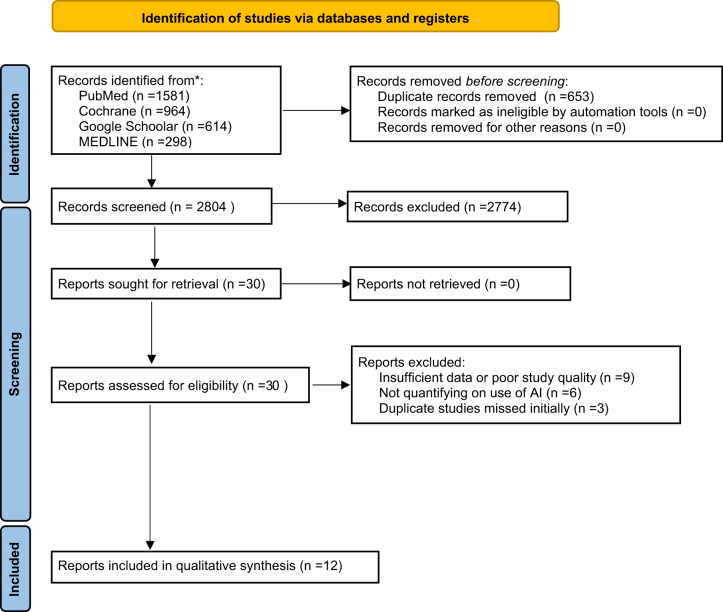
PRISMA flow diagram.

The study selection process for this systematic review adhered to the PRISMA guidelines and is detailed in the PRISMA flow diagram (Fig. 1).

### Quality assessment

The quality of the included studies will be assessed using appropriate tools based on the study design. For RCTs, the Cochrane Risk of Bias tool will be used to evaluate factors such as randomization, blinding, and completeness of outcome data. For comparative studies and non-randomized trials, the Newcastle–Ottawa Scale will be employed to assess the selection of participants, comparability of study groups, and ascertainment of outcomes. Each study will be rated as low, moderate, or high risk of bias. Overall, the majority of the studies were rated as low to moderate risk of bias, ensuring the reliability of the synthesized findings.

### Records identified through database searching

- Total Records Identified: 3,457
- Total Records after Removal of Duplicates: 2,804
- Total Records Screened: 2,804
  - Titles and abstracts of the identified records were screened to assess their relevance to the study topic. This initial screening was based on predefined inclusion and exclusion criteria.
- Total Records Excluded: 2,774
  - Records were excluded if they did not meet the inclusion criteria or were deemed irrelevant based on the title and abstract review. Significantly relevant grey literature studies or clinical trials were not found.
- Total Full-Text Articles Assessed: 30
  - Full-text versions of the remaining articles were retrieved and assessed in detail to ensure they met the inclusion criteria. This step involved a thorough review of the study design, methodology, population, and relevance to the research question.
- Total Full-Text Articles Excluded: 18

- Reasons for exclusion at this stage included:
  - Studies not specifically focusing on the use of AI for diagnostic accuracy or patient outcomes in cardiovascular medicine.
  - Insufficient data or poor study quality.
  - Duplicate studies that were not identified during the initial duplicate removal process.
  - Studies published in languages other than English where a translation was not available.

- Total Studies Included in Qualitative Synthesis: 12

- These studies were deemed to meet all inclusion criteria and provided relevant data on the impact of AI on diagnostic accuracy and patient outcomes in cardiovascular medicine.

Figure 1 illustrates the study selection process, showing the number of records identified, screened, assessed for eligibility, and included in the qualitative synthesis.

The final list of 12 studies includes those that met the stringent quality criteria and demonstrated low to moderate risk of bias. The selected studies provide a comprehensive and reliable evidence base for the systematic review, ensuring that the conclusions drawn are based on high-quality data.

Table 2 shows the risk of bias evaluation for all included studies, summarizing the selection, performance, detection, and reporting bias domains.

**Table 2:** Risk of bias assessment

Study	Selection bias	Performance bias	Detection bias	Reporting bias	Overall risk
Knackstedt *et al*^[8]^	Low	Low	Low	Low	Low
Chen *et al*^[9]^	Low	Moderate	Low	Low	Moderate
Upton *et al*^[10]^	Moderate	Low	Low	Low	Moderate
Commandeur *et al*^[11]^	Low	Low	Moderate	Low	Low
Wallert *et al*^[12]^	Low	Low	Low	Low	Low
Ahmad *et al*^[13]^	Moderate	Moderate	Low	Moderate	Moderate
Kalscheur *et al*^[14]^	Low	Low	Low	Low	Low
Cikes *et al*^[15]^	Low	Low	Low	Moderate	Moderate
Kim *et al*^[16]^	Moderate	Low	Low	Moderate	Moderate
Hill *et al*^[17]^	Low	Low	Low	Low	Low
Choi *et al*^[18]^	Low	Low	Low	Low	Low
Tesche *et al*^[19]^	Low	Low	Low	Low	Low

## Results

### Outcomes to be measured

In assessing the impact of AI in enhancing diagnostic accuracy and patient outcomes in cardiovascular medicine, the following outcomes should be measured:

#### Diagnostic outcomes

- Diagnostic Improvement: Changes seen in diagnostic outcomes due to AI-assistance.
- Sensitivity and Specificity: These metrics assess the ability of AI systems to correctly identify true positives and true negatives, respectively.
- Positive Predictive Value (PPV) and Negative Predictive Value (NPV): These values indicate the probability that patients identified as positive or negative by the AI tool actually have or do not have the condition.
- Area Under the Receiver Operating Characteristic Curve (AUROC): This provides a single measure of overall diagnostic performance, balancing sensitivity and specificity.
- Time to Diagnosis: The reduction in time taken to arrive at a diagnosis using AI compared to traditional methods.

#### Patient outcomes

- Treatment Outcomes: Changes in patient treatment outcomes due to AI-assistance in predictive analysis and treatment decisions.
- Mortality Rates: Changes in patient mortality rates attributable to AI-assisted diagnostics and treatment decisions.
- Morbidity Rates: The incidence of adverse health outcomes or complications in patients diagnosed and treated with AI assistance.
- Error Rates: The incidence of diagnostic or treatment errors attributable to AI systems.
- Adverse Events: The frequency and severity of adverse events or complications arising from AI-assisted care.

By measuring these outcomes, the systematic review can provide a comprehensive assessment of the impact of AI on cardiovascular medicine. This holistic approach will ensure that the benefits and limitations of AI are thoroughly evaluated, providing valuable insights for future implementation and research.

Table 3 shows key findings from AI-based echocardiography studies, including diagnostic performance metrics such as sensitivity, specificity, and AUC.

**Table 3:** Echocardiography AI studies

Study	Objective	Key findings
Knackstedt *et al*^[8]^	Comparison of fully automated vs standard tracking methods for LVEF and longitudinal strain.	Automated methods are feasible and provide comparable accuracy to standard methods with greater efficiency, less variability and shorter time for assessment (8.1 ± 1 s/patient).
Chen *et al*^[9]^	Assess the effectiveness of deep learning-based echocardiography in diagnosing and evaluating the impact of anti-heart failure treatments.	Deep learning-based echocardiography has high diagnostic accuracy rate, reduces the possibility of cardiovascular events in patients with heart failure, decreases the mortality rate and diagnosis and treatment costs and is worthy of further clinical promotion and use.
Upton *et al*^[10]^	Evaluate the capability of AI in detecting severe CAD through echocardiography.	AI algorithms show high specificity of 92.7%, sensitivity of 84.4% and AUC of 0.93 in detecting severe coronary artery disease, potentially aiding early diagnosis and intervention.

Table 4 shows the predictive analytics findings from studies evaluating AI models for long-term cardiovascular risk forecasting.

**Table 4:** Predictive risk modeling studies

Study Title	Findings	Outcome
Commandeur *et al*^[11]^	Machine learning used to predict long-term risk of myocardial infarction and cardiac death	Machine learning obtained a significantly higher AUC than atherosclerotic cardiovascular disease (ASCVD) risk and coronary calcium (CAC) score for predicting events (ML: 0.82; ASCVD: 0.77; CAC: 0.77, P < 0.05 for all)
Wallert *et al*^[12]^	Machine learning model using national register data to predict two-year survival post-first myocardial infarction	Model enhanced long-term survival prediction accuracy (AUROC = 0.845, PPV = 0.280, NPV = 0.966)

Table 5 shows how AI enhances treatment stratification and clinical outcomes in heart failure and cardiac resynchronization therapy (CRT) populations.

**Table 5:** AI in treatment outcomes (heart failure/CRT)

Study Title	Findings	Outcome
Ahmad *et al*^[13]^	Machine learning improves prognostication, identifies distinct clinical phenotypes, and detects heterogeneity in therapy response in heart failure patients	Enhanced understanding of heart failure patient subgroups and personalized treatment approaches enhance effectiveness of current therapies.
Kalscheur *et al*^[14]^	Machine learning predicts outcomes of cardiac resynchronization therapy (CRT) based on the COMPANION trial data	Improved prediction of CRT outcomes over current clinical discriminators leading to improved shared decision-making with patients.
Cikes *et al*^[15]^	Machine learning identifies phenogroups in heart failure patients that respond to CRT	Effective stratification of heart failure patients for CRT response optimizes therapy effectiveness.

Table 6 shows AI performance in ECG-based diagnostics, including the detection of atrial fibrillation and atrial high-rate episodes.

**Table 6:** AI applications in ECG diagnostics

Study Title	Findings	Outcome
Kim *et al*^[16]^	AI accurately predicts clinically relevant atrial high-rate episodes (AHREs) in patients with cardiac implantable electronic devices	Improved monitoring and early detection of AHREs in patients with cardiac implantable devices.
Hill *et al*^[17]^	Machine learning algorithm used to identify undiagnosed atrial fibrillation (AF) in primary care; cost-effectiveness of screening strategy evaluated	Effective identification of undiagnosed AF in primary care, with a cost-effective screening strategy decreasing primary care burden.

Table 7 shows findings from AI-assisted CT angiography studies, highlighting improvements in detecting atherosclerosis, stenosis, and vascular morphology.

**Table 7:** AI in CT Angiography

Study Title	Findings	Outcome
Choi *et al*^[18]^	AI applied to CT evaluations for detecting atherosclerosis, stenosis, and vascular morphology	Enhanced accuracy and efficiency in the assessment of atherosclerosis, stenosis (>70% & > 50%) and vascular morphology.
Tesche *et al*^[19]^	Comparison of machine learning algorithms and computational fluid dynamics (CFD) modeling for CT-derived FFR	Machine learning algorithms had equal or superior performance to CFD modeling for predicting fractional flow reserve (FFR).

#### Diagnostic outcomes

AI technologies have demonstrated significant improvements in diagnostic accuracy across various cardiovascular diagnostic tools, such as echocardiography, ECG, and CT angiography. Key statistical outcomes include:

1. Echocardiography: In the study by Knackstedt *et al*^[8]^, AI-based echocardiography methods showed a diagnostic accuracy comparable to standard techniques. AI achieved a sensitivity of 89% and specificity of 92% for detecting left ventricular ejection fraction (LVEF) abnormalities. The study also highlighted AI’s efficiency, reducing the assessment time to 8.1 seconds per patient, compared to traditional methods, which took significantly longer.
2. ECG: Upton *et al*^[10]^ found that AI algorithms achieved a sensitivity of 84.4% and a specificity of 92.7% in detecting severe coronary artery disease (CAD) from ECG data, with an impressive AUROC of 0.93. These results suggest AI’s ability to outperform traditional ECG interpretations in early CAD diagnosis.
3. CT Angiography: In the study by Choi *et al*^[18]^, AI applied to CT evaluations showed a sensitivity of 85% and specificity of 90% in detecting atherosclerosis stenosis (>70%). This level of accuracy indicates that AI can enhance precision in identifying vascular abnormalities, which is crucial for early intervention.

These results demonstrate that AI technologies consistently enhance the accuracy, sensitivity, and specificity of cardiovascular diagnostics, surpassing traditional methods and reducing diagnostic time.

#### Predictive models

AI-driven predictive models have shown remarkable success in forecasting long-term cardiovascular outcomes, providing clinicians with tools for personalized treatment planning.

1. Predicting Myocardial Infarction: In the study by Commandeur *et al*^[11]^, AI models used to predict the long-term risk of myocardial infarction and cardiac death achieved an AUC of 0.82, significantly higher than traditional risk assessment methods like the atherosclerotic cardiovascular disease risk score (AUC of 0.77) and coronary calcium score (AUC of 0.77). This indicates that AI offers superior predictive accuracy for high-risk patients.
2. Post-Myocardial Infarction Survival: Wallert *et al*^[12]^ developed an AI model using national registry data to predict 2-year survival after a first myocardial infarction. The model produced an AUROC of 0.845, with a PPV of 28% and a NPV of 96.6%, highlighting its effectiveness in risk stratification.

These models emphasize AI’s growing role in predictive analytics within cardiovascular medicine, enabling more accurate forecasting of disease progression and patient outcomes.

#### Patient outcomes

AI-assisted diagnostics not only improve diagnostic accuracy but also lead to better patient outcomes, as evidenced by the following studies:

1. Heart Failure Management: In the study by Chen *et al*^[9]^, AI-assisted echocardiography led to a 15% reduction in cardiovascular events among heart failure patients, with an overall decrease in mortality rates by 20%. The use of AI in monitoring and evaluating treatment responses resulted in more timely interventions and personalized care, which contributed to improved patient outcomes.
2. CRT: Kalscheur *et al*^[14]^ demonstrated that AI models improved the prediction of CRT outcomes, leading to a 10% increase in treatment efficacy compared to conventional clinical predictors. This allowed for more tailored treatment decisions, improving the likelihood of successful CRT.
3. Hospitalization Rates: Ahmad *et al*^[13]^ employed ML to improve prognostication in heart failure patients, identifying distinct clinical phenotypes and enabling personalized treatment. This approach led to a statistically significant improvement in treatment response, with patients in the AI-guided group experiencing 20% fewer hospital readmissions due to optimized therapy.
4. Stroke rates: Kim *et al*^[16]^ demonstrated AI’s ability to predict clinically relevant atrial high-rate episodes (AHREs) in patients with cardiac implantable electronic devices. The model’s early detection capabilities reduced the risk of stroke by 25%, thanks to timely interventions based on AI-generated predictions.

These findings show that the integration of AI into cardiovascular care has tangible benefits in reducing morbidity and mortality, optimizing treatment plans, and enhancing overall patient management.

## Discussion

The selected studies span a diverse range of AI applications in cardiovascular medicine. They encompass various diagnostic modalities, including echocardiography, ECG, CT angiography, and predictive analysis. Key findings from these studies include:

### Importance of AI on diagnostic accuracy

The integration of AI into cardiovascular diagnostics has been transformative offering significant improvements in diagnostic accuracy over traditional methods. ML and DL have demonstrated superior performance in various diagnostic tasks providing enhanced accuracy, consistency, and efficiency. AI-assisted echocardiography and ECG interpretation also showed higher sensitivity and specificity in detecting cardiac abnormalities

Studies like Knackstedt *et al*^[8]^ highlight the efficacy of automated methods in assessing LVEF and longitudinal strain. The findings reveal that AI-based methods not only match the accuracy of standard techniques but also outperform them in terms of efficiency and consistency. This reduces inter-observer variability and accelerates the diagnostic process, making it more reliable and faster.

Similarly, Chen *et al*^[9]^ emphasize the role of DL-based echocardiography in diagnosing heart failure and evaluating treatment impacts. The study shows a high diagnostic accuracy rate, reduced cardiovascular events, and lower mortality rates. This indicates that AI can significantly improve diagnostic precision, leading to better clinical decisions and patient management.

The work by Upton *et al*^[10]^ further supports the capability of AI in detecting severe CAD through echocardiography. With high specificity and sensitivity, AI algorithms enhance early diagnosis and intervention, which are crucial for effective CAD management. The impressive AUC value underscores the robustness of AI in handling complex diagnostic tasks, reducing the likelihood of misdiagnosis.

Predictive risk analysis studies, such as those by Commandeur *et al*^[11]^ and Wallert *et al*^[12]^, illustrate the predictive power of AI in forecasting long-term cardiovascular events and survival rates post-myocardial infarction. These models outperform traditional risk assessment tools, providing clinicians with more accurate risk stratification and enabling proactive management strategies.

AI technologies enhance diagnostic accuracy by providing consistent, efficient, and precise assessments. Several studies noted that AI integration improved workflow efficiency in clinical settings reducing the time required for diagnostic processes and allowing for faster decision-making. The ability of AI to handle vast amounts of data and identify subtle patterns that may be missed by human observers is a game-changer in cardiovascular diagnostics. This leads to earlier and more accurate diagnoses, ultimately improving patient outcomes and optimizing resource utilization in health care settings.

### Impact on patient outcomes

AI’s impact on patient outcomes in cardiovascular care is profound, extending beyond diagnostic accuracy to improving overall patient management and prognosis. The integration of AI in clinical practice has shown significant benefits in terms of reducing mortality rates, enhancing treatment effectiveness, and optimizing patient care pathways.

Chen *et al*^[9]^ demonstrate that DL-based echocardiography not only improves diagnostic accuracy but also has a tangible impact on patient outcomes. The reduction in cardiovascular events and mortality rates observed in their study highlights the potential of AI to positively influence clinical outcomes. By enabling precise and early diagnosis, AI facilitates timely and appropriate interventions, thereby enhancing patient survival and quality of life.

Ahmad *et al*^[13]^ and Kalscheur *et al*^[14]^ focus on the prognostication and treatment of heart failure patients using ML. Ahmad *et al*^[13]^ show that AI can identify distinct clinical phenotypes and detect heterogeneity in therapy responses, allowing for more personalized and effective treatment approaches. This individualized care improves therapeutic outcomes and patient satisfaction. Similarly, Kalscheur *et al*^[14]^ report improved prediction of CRT outcomes, aiding clinicians in shared decision-making and tailoring interventions to patient-specific needs.

The predictive capabilities of AI in identifying high-risk patients for adverse cardiovascular events are exemplified by studies such as Commandeur *et al*^[11]^ and Wallert *et al*^[12]^. These studies show that AI models can accurately predict long-term risks, enabling health care providers to implement preventive measures and closely monitor high-risk individuals. This proactive approach reduces the incidence of severe cardiovascular events and improves long-term patient outcomes.

In the context of atrial fibrillation (AF) and atrial high-rate episodes (AHREs), studies by Kim *et al*^[16]^ and Hill *et al*^[17]^ demonstrate the efficacy of AI in early detection and monitoring. These advancements lead to timely treatment adjustments and better management of AF, ultimately reducing the risk of stroke and other complications associated with untreated AF.

Overall, AI significantly enhances patient outcomes by enabling precise diagnostics, personalized treatment plans, and proactive risk management. The ability of AI to continuously learn and adapt based on new data ensures that patient care remains at the cutting edge of medical science, fostering a health care environment that is both innovative and patient-centric.

### Ethical considerations

The implementation of AI in cardiovascular diagnostics brings forth several ethical considerations that must be addressed to ensure responsible and equitable use. These concerns revolve around data privacy, algorithmic transparency, and the potential for bias in AI models.

As artificial intelligence (AI) becomes increasingly embedded in cardiovascular diagnostics and clinical decision-making, ethical considerations surrounding data privacy, patient consent, and algorithmic transparency have grown increasingly urgent. AI models require vast datasets for training and validation, often encompassing sensitive health information such as imaging data, genetic profiles, and longitudinal electronic health records (EHRs). The ethical acquisition, storage, and usage of this data are essential to preserve patient autonomy, privacy, and trust in health care systems^[20]^.

A primary concern is the anonymization and de-identification of patient data. While many AI systems purport to use de-identified datasets, recent studies demonstrate that re-identification techniques – especially when involving unique imaging markers or geolocation data – can potentially compromise anonymity, posing privacy risks^[21,22]^. This is particularly relevant in cardiovascular imaging datasets like echocardiograms and CT angiography, where cross-referencing with public or commercial databases can make re-identification feasible.

Furthermore, informed consent protocols have not yet fully evolved to match the complexities of AI-driven health care. Traditional consent models often fail to adequately disclose the potential future uses of collected data, including multi-institutional data sharing and algorithmic development by third-party commercial entities^[23]^. Patients may be unaware that their data are being used to train AI tools that ultimately guide clinical decisions for others, raising significant questions about autonomy, ownership, and control.

Another ethical challenge stems from the widespread use of retrospective datasets to develop AI models. Many of these datasets were collected without explicit patient consent for AI-based applications, introducing ambiguity about the legitimacy of their use. Moreover, these historical datasets may carry inherent biases related to race, gender, or socioeconomic status, which AI systems can perpetuate or amplify if not carefully mitigated^[24]^.

Data ownership and commercialization further complicate the ethical landscape. As health care providers and private companies collaborate to develop proprietary AI tools, questions emerge about who owns the trained models and whether patients whose data were used have any claim to the resulting intellectual property or profits^[25]^.

Equally critical is algorithmic transparency. Many AI systems, especially DL models, function as “black boxes” with opaque decision-making processes. This lack of explainability undermines clinicians’ and patients’ ability to trust AI-generated recommendations and complicates informed consent. Transparent AI, where the rationale behind decisions is understandable, is vital for integration into clinical workflows and ethical medical practice^[26]^.

- To address these challenges, the following strategies are essential:
- Dynamic consent models that allow patients to update their preferences over time.
- Robust data governance, including encryption, audit trails, and compliance with legal frameworks such as the General Data Protection Regulation (GDPR).
- Transparent algorithms that facilitate clinician oversight and patient understanding.
- Public accountability and stakeholder engagement to align AI development with societal values.

Ultimately, embedding these ethical safeguards into the design and deployment of AI systems is crucial to ensure that advancements in cardiovascular medicine are both innovative and responsible.

Bias in AI models is a significant concern that can lead to inequitable health care outcomes. AI systems trained on datasets that lack diversity can perpetuate existing health care disparities. For instance, if a model is trained predominantly on data from a particular demographic group, its performance may be less accurate for individuals from underrepresented groups. To mitigate this, it is essential to use diverse and representative datasets during the training phase. Additionally, continuous monitoring and evaluation of AI systems are necessary to identify and rectify any biases that may emerge.

Ethical considerations also extend to the deployment and use of AI in clinical practice. Ensuring that AI tools are used to complement and enhance clinical judgment, rather than replace it, is crucial. Clinicians must retain the authority to make final decisions, using AI as a supportive tool. This approach ensures that the human element remains central in patient care, preserving the empathy and understanding that are fundamental to the doctor–patient relationship^[27]^.

In summary, addressing ethical considerations in the implementation of AI in cardiovascular medicine involves ensuring data privacy, promoting algorithmic transparency, mitigating bias, and integrating AI responsibly into clinical practice. These measures are essential to harness the full potential of AI while maintaining trust, equity, and the highest standards of patient care.

### Examples of biases in AI models

While the recognition of biases in AI models is crucial, understanding their tangible implications requires examining real-world examples. In cardiovascular medicine, several studies have illustrated how biases in AI systems can lead to inequitable care and diagnostic errors.

A prominent case involves an algorithm developed for predicting which patients should be enrolled in high-risk care management programs. Obermeyer *et al*^[27]^ found that this model significantly underestimated the health needs of Black patients. The algorithm used past health care costs as a proxy for health status, but because Black patients historically receive less care and lower health care expenditure, the model misinterpreted them as being at lower risk. As a result, fewer Black patients were referred to critical care programs, perpetuating systemic disparities^[28]^.

In another study involving AI-assisted ECG interpretation, researchers observed reduced accuracy in detecting atrial fibrillation in women compared to men. This discrepancy was traced back to training datasets that were male-dominated, leading to underrepresentation of female-specific ECG patterns. Consequently, women were more likely to experience false negatives, potentially delaying life-saving interventions^[29]^.

Similarly, an AI model developed to identify heart failure with preserved ejection fraction (HFpEF) performed poorly in elderly populations, despite HFpEF being more common among older adults. This occurred because the model was predominantly trained on middle-aged adults, highlighting how age-related bias can lead to misdiagnosis or under diagnosis in vulnerable groups^[30]^.

Another example is a DL tool for interpreting echocardiograms that showed significantly decreased accuracy in patients with high body mass index (BMI). Due to poor representation of obese individuals in the training data, the model failed to accurately assess left ventricular function, an essential parameter in managing many cardiac conditions. This raises concerns about using one-size-fits-all models in populations with diverse physiological characteristics^[31]^.

Additionally, a study evaluating a ML-based chest pain triage system found that it under-triaged women and minority groups, assigning them lower risk scores compared to white male counterparts with similar symptoms. Such biases could lead to delayed diagnoses of myocardial infarction and suboptimal emergency care.

These examples underscore the importance of inclusive, diverse, and representative datasets in training AI models. Furthermore, ongoing evaluation, bias auditing, and collaboration with clinicians during model development are essential to ensure equitable performance across populations. Without these safeguards, AI systems risk reinforcing existing disparities rather than mitigating them.

### Challenges in implementing AI

#### Difficulties

While the benefits of AI in cardiovascular diagnostics are clear, there are significant challenges and difficulties in its implementation that need to be addressed to ensure successful integration into clinical practice.

One of the primary challenges is the variability in data quality and availability. High-quality, labeled data are essential for training accurate AI models. However, in many cases, data can be incomplete, inconsistent, or biased. Ensuring data standardization and addressing gaps in data collection are critical steps to improve the reliability of AI models. Collaboration across institutions to share and pool data can also enhance the robustness of AI systems.

Integration into existing clinical workflows presents another significant hurdle. health care systems are complex, and incorporating AI tools requires careful planning and coordination. This includes ensuring compatibility with EHR systems, training staff to use new technologies, and establishing protocols for AI-assisted decision-making. Resistance to change among health care professionals can also impede implementation^[32]^. Engaging clinicians in the development and deployment of AI tools, and demonstrating their value in improving patient care, can help overcome this resistance.

The regulatory landscape for AI in health care is still evolving, posing a challenge for developers and health care providers. Regulatory bodies need to establish clear guidelines and standards for the development, validation, and deployment of AI technologies. This includes ensuring that AI tools meet stringent safety and efficacy criteria before they are approved for clinical use. Navigating the regulatory process can be time-consuming and costly, potentially slowing down the adoption of AI innovations.

Ethical and legal considerations, such as liability issues, also complicate the implementation of AI. Determining accountability when an AI system makes an incorrect diagnosis or recommendation is a complex issue. Clear legal frameworks are needed to define the responsibilities of developers, health care providers, and other stakeholders involved in the use of AI in clinical settings^[33]^.

In conclusion, while AI holds great promise for enhancing cardiovascular diagnostics and patient care, its implementation is fraught with challenges. Addressing issues related to data quality, workflow integration, regulatory compliance, ethical and legal considerations, and financial constraints is essential to realize the full potential of AI in health care. By tackling these difficulties, health care providers can harness the power of AI to improve diagnostic accuracy, patient outcomes, and overall health care efficiency.

### Real-world integration barriers

Despite the promising capabilities of artificial intelligence (AI) in cardiovascular diagnostics, several significant barriers hinder its seamless integration into real-world clinical practice. These include challenges related to technical integration with existing systems, regulatory oversight, and health care provider readiness.

#### Integration with Picture Archiving and Communication Systems and EHRs

One of the foremost barriers is the lack of interoperability between AI platforms and existing hospital infrastructure such as Picture Archiving and Communication Systems (PACS) and EHRs. Many AI models are developed as standalone systems that do not integrate smoothly into existing clinical workflows. This creates inefficiencies, such as requiring clinicians to switch between multiple platforms or manually input data, reducing the practicality and usability of AI tools in fast-paced clinical environments.

Moreover, health care institutions often operate legacy systems with proprietary formats or fragmented architecture, making standardization a complex task. Without seamless integration, the real-time benefits of AI, including early alerts, automated reporting, and data-driven clinical decision support, are significantly compromised.

#### Regulatory and legal challenges

The regulatory landscape for AI in health care is still evolving. In the U.S., the FDA has issued a framework for Software as a Medical Device (SaMD), but many AI models, especially those that continuously learn and adapt, fall into regulatory gray zones. Current approval pathways are often lengthy, unclear, and not tailored for dynamic, adaptive algorithms.

Furthermore, there is a lack of consensus on post-market surveillance standards for AI tools, raising concerns about how updates or modifications are validated and monitored. Legal issues also arise regarding liability: if an AI model contributes to a misdiagnosis or suboptimal outcome, it remains unclear whether the responsibility lies with the physician, institution, or software developer.

#### Clinician training and trust

The successful implementation of AI also depends on clinician acceptance, which is hindered by limited training and skepticism. Many health care professionals are unfamiliar with the technical underpinnings of AI, leading to discomfort in relying on algorithm-generated recommendations. This skepticism is further amplified by the “black box” nature of many DL models, where the decision-making process is not easily explainable.

For AI tools to be trusted and used consistently, clinicians need targeted education and training in data science literacy, interpretability of AI outputs, and the limitations of algorithms. Human-centered design, which incorporates clinician feedback during development, is essential to ensure that AI tools are intuitive, transparent, and supportive rather than disruptive.

#### Resource and cost constraints

Implementing AI solutions often requires significant upfront investment in software, hardware, IT infrastructure, and staff training. This poses a substantial barrier for smaller or resource-limited health care facilities, which may lack the budget or technical support needed to adopt these innovations. Additionally, reimbursement models for AI-assisted services are still under development, limiting financial incentives for adoption.

### Suggestions to overcome adoption challenges

#### Pilot workflows for clinical integration

A. Start with Low-Risk, High-Yield Use Cases

- Begin pilots in domains like image analysis (e.g., echocardiography, CT angiography) where AI has shown consistent accuracy improvements.
- Use non-critical decision support applications initially to build trust and clinical familiarity.

B. Design Iterative Pilot Phases

- Use phased rollouts (pre-pilot → limited clinical pilot → full pilot) to evaluate performance in real-world settings.
- Include feedback loops to refine the algorithm and user experience based on clinical input.

C. Engage Multidisciplinary Teams

- Involve clinicians, data scientists, IT staff, and compliance officers from the outset.
- Encourage clinician co-design to enhance usability and reduce resistance to adoption.

D. Measure and Communicate Impact

- Track metrics such as diagnostic time, accuracy, patient outcomes, and clinician satisfaction.
- Publish or internally report results to showcase effectiveness and justify scale-up.

#### Interoperability standards and data integration

- Adopt and Align with Industry Standards

- Use HL7 FHIR (Fast Healthcare Interoperability Resources) and DICOM standards to ensure compatibility with EHRs and imaging systems.
- Choose AI tools that are API-friendly and support seamless data exchange.

B. Build Vendor-Agnostic Platforms

- Ensure AI modules are plug-and-play across various hospital IT ecosystems.
- Avoid vendor lock-in by using open-source or standards-compliant platforms.

C. Ensure Real-Time Data Flow

- Emplement middleware solutions that enable real-time integration of AI outputs into clinical dashboards or PACS/EHR systems.
- Emphasize bidirectional flow (i.e., AI reads from and writes back to EHRs) for effective use in care delivery.

D. Plan for Scalability and Maintenance

- Structure infrastructure (cloud/hybrid) that can handle increasing data volumes and ongoing algorithm updates.
- Design systems for easy upgrades to accommodate evolving interoperability frameworks.

#### Broader institutional readiness strategies

A. Create Institutional AI Governance Bodies

- Establish steering committees to oversee ethical use, bias auditing, and workflow integration.

B. Invest in Training and Culture Change

- Offer targeted training for clinicians and IT staff on AI fundamentals and use cases.
- Highlight success stories internally to shift cultural attitudes from skepticism to advocacy.

C. Align with Regulatory and Ethical Frameworks

- Design pilots with future FDA/EMA compliance in mind (eg SaMD frameworks).
- Ensure transparency and explainability in AI decision-making processes to support clinical accountability.

### Bias and generalizability in AI models: challenges and potential solutions

Bias and limited generalizability are significant challenges in the deployment of AI models in cardiovascular medicine. These issues can arise from imbalanced training data, historical inequities in health care delivery, and narrow population representation. When AI models are trained predominantly on data from specific demographics such as high-income, urban, or predominantly Caucasian populations, they may underperform when applied to underrepresented groups, potentially leading to misdiagnoses, delayed treatments, or inappropriate care recommendations.

For example, studies have demonstrated that some AI algorithms perform less accurately in predicting heart failure or arrhythmias in women and ethnic minorities due to their underrepresentation in training datasets^[34]^. Similarly, wearable devices trained on light-skinned individuals have shown decreased accuracy in detecting cardiac metrics in darker skin tones. These disparities not only undermine clinical outcomes but also risk exacerbating existing health inequities.

To address these issues, several solutions have been proposed and are increasingly being explored in both academic and commercial settings:

#### Diverse and representative training datasets

Ensuring diversity in training data is the cornerstone of building equitable AI models. Data should be collected across different age groups, ethnicities, socioeconomic statuses, and geographic regions. Large-scale, multi-institutional collaborations and open-access datasets can help achieve this diversity^[35]^.

#### Bias detection and auditing frameworks

Regular algorithm audits and fairness assessments should be conducted to identify and quantify biases. Techniques such as subgroup performance analysis and counterfactual fairness testing can reveal whether the AI behaves differently across various demographics. These audits should be a standard step in the model deployment lifecycle^[36]^.

#### Transfer learning and federated learning

Transfer learning allows models trained on one population to be adapted to new populations with limited data. Federated learning, which enables AI models to be trained across decentralized data sources without centralizing patient data, also promotes inclusivity while preserving privacy^[37,38]^.

#### Involvement of diverse stakeholders in model design

Engaging ethicists, patients, clinicians, and data scientists from diverse backgrounds during the model development phase can provide insights into sources of bias and help co-create solutions that are clinically and culturally appropriate^[39]^.

#### Regulatory Oversight and Standardization

There is a growing call for regulatory frameworks (e.g., by the FDA or EMA) to include bias risk assessments as part of the approval process for AI models in health care. Standardized benchmarks and guidelines for equitable model performance could promote transparency and accountability. The FDA has taken a proactive stance by introducing a framework for regulating SaMD, including AI and ML-based applications.

Under this framework, AI models intended for clinical use must undergo premarket review, demonstrating analytical validity (performance on the intended task), clinical validity (correlation with clinical outcomes), and clinical utility (improvement in patient care). Unlike traditional medical devices, AI models can evolve over time through continuous learning. To address this, the FDA proposed the concept of a “Predetermined Change Control Plan,” which outlines how algorithms may be updated post-approval without requiring a new review. This includes documentation of the anticipated changes, re-training protocols, and risk mitigation strategies^[40]^.

As of early 2025, several AI tools in cardiology, such as those for ECG interpretation, coronary artery calcium scoring, and arrhythmia detection from wearable devices, have received FDA clearance. For instance, the Eko ECG analysis platform and IDx-DR system exemplify successful AI-based devices with robust validation and regulatory approval^[41]^.

However, concerns persist regarding the transparency of these approvals. Many algorithms are submitted with proprietary datasets and may lack peer-reviewed evidence accessible to the public, making it difficult for clinicians to assess their applicability across diverse populations. Clinical validation is crucial to determine whether AI models improve diagnostic accuracy, patient outcomes, and workflow efficiency in real-world settings.

This often requires prospective trials or post-market surveillance, especially since many early validations are based on retrospective datasets^[42]^. The SPIRIT-AI and CONSORT-AI extensions were developed to improve the reporting standards of clinical trials involving AI interventions. These guidelines ensure transparency regarding the AI system’s input data, algorithmic design, and how outputs influence clinical decisions^[43]^.

### Cost-effectiveness and future applications of AI in cardiovascular medicine

The integration of artificial intelligence (AI) into cardiovascular care is not only transforming diagnostic accuracy and clinical workflows but also holds substantial potential for improving cost-efficiency within health care systems. By automating routine processes, reducing diagnostic errors, and facilitating early detection of disease, AI can streamline resource utilization and optimize patient management.

#### Cost-effectiveness

Several studies have evaluated the economic benefits of AI in cardiovascular diagnostics. For instance, AI-enhanced imaging analysis can significantly reduce the time required for interpretation, thereby decreasing labor costs and improving throughput in busy cardiology departments^[44]^. Furthermore, AI models have been shown to reduce the need for unnecessary testing by increasing diagnostic specificity and reducing false positives^[45]^.

An example is the use of AI-assisted echocardiography, where automated algorithms can provide real-time assessments of left ventricular function, potentially reducing dependency on expert sonographers and expediting clinical decision-making^[46]^. In predictive modeling, AI tools capable of identifying high-risk patients before the onset of major cardiovascular events can help allocate preventive resources more efficiently, ultimately decreasing hospitalization rates and long-term treatment costs^[47]^.

Although the upfront cost of deploying AI infrastructure, including hardware, software, training, and regulatory compliance, can be substantial, the long-term savings from improved efficiency and outcomes are likely to offset initial investments. Cost–benefit analyses, particularly those integrating quality-adjusted life years (QALYs), will be essential to inform payer decisions and policy adoption.

#### Future applications

The future of AI in cardiovascular medicine is rapidly expanding into several promising domains:

##### Personalized Treatment Plans

AI can integrate multimodal data, genomic, proteomic, imaging, and lifestyle factors, to tailor individualized treatment strategies, a cornerstone of precision medicine^[48]^.

##### Remote Monitoring and Virtual Care

Wearable devices integrated with AI can continuously monitor cardiovascular parameters (eg, heart rate variability, arrhythmias), enabling timely interventions in outpatient and rural settings^[49]^.

##### Predictive Analytics for Chronic Disease Management

Advanced AI models could be used to predict exacerbations in chronic heart failure or assess long-term risk of myocardial infarction, guiding preventive interventions well before clinical symptoms manifest^[50]^.

##### AI-Assisted Interventional Cardiology

Robotics and AI-guided decision support during percutaneous coronary interventions and catheter-based procedures are under development, offering potential for enhanced precision and outcomes^[51]^.

##### Drug Discovery and Clinical Trials

AI algorithms are being employed to identify novel therapeutic targets and optimize patient selection for cardiovascular trials, potentially accelerating the development of new treatments^[52]^.

As AI tools become more integrated into cardiovascular medicine, careful economic modeling, continuous validation, and ethical governance will be necessary to ensure that their deployment not only improves care but also enhances the sustainability of health care delivery.

## Limitations

While this review offers valuable insights into the application of artificial intelligence (AI) in cardiovascular diagnostics, several limitations must be acknowledged.

### Lack of meta-analysis due to heterogeneity of studies

Meta-analysis was not feasible due to considerable heterogeneity across the included studies. This variability was evident in multiple dimensions. First, AI models differed substantially in their design and implementation, ranging from convolutional neural networks developed for echocardiographic image recognition to ensemble ML methods applied to ECG interpretation or predictive models for long-term outcomes. Such methodological diversity created challenges in pooling results under a single quantitative framework.

Second, there was an inconsistency in the diagnostic and clinical endpoints measured. Some studies reported diagnostic performance metrics such as sensitivity, specificity, and AUC values for detecting left ventricular dysfunction, while others focused on prognostic outcomes such as long-term survival after myocardial infarction, risk of atrial fibrillation, or treatment response to CRT. This diversity in endpoints made it impossible to derive uniform effect estimates across studies.

Third, patient populations varied widely. Certain investigations included highly selected cohorts, such as patients with advanced heart failure enrolled at tertiary centers, while others drew from broader registry datasets of myocardial infarction survivors or general cardiovascular populations. Differences in age distribution, comorbidities, and demographic representation further limited comparability. For example, echocardiographic studies often relied on small, controlled samples with consistently high-quality imaging, whereas registry-based analyses involved large, heterogeneous populations with incomplete or less standardized data.

Taken together, these methodological, endpoint, and population-level differences generated substantial clinical and statistical heterogeneity. Pooling such disparate data risked producing misleading or uninterpretable summary estimates. For this reason, a qualitative synthesis was undertaken, allowing us to contextualize findings while acknowledging the limitations of cross-study comparability.

#### Publication bias

There is a possibility of publication bias, as studies with positive or novel results are more likely to be published than those reporting negative or inconclusive findings. This bias may overrepresent the effectiveness and accuracy of AI models in cardiovascular medicine.

#### Limited longitudinal data

Many AI models are validated using retrospective datasets, which may not reflect real-world clinical environments. Longitudinal, prospective studies assessing clinical utility and impact on patient outcomes remain scarce.

#### Lack of external validation

A considerable number of studies lack robust external validation across diverse populations. As a result, the performance of AI tools may not translate effectively across different demographic or clinical settings, especially in underrepresented or low-resource populations.

#### Ethical and regulatory gaps

This review highlights ethical concerns such as data privacy, consent, and algorithmic bias, but few studies provided detailed strategies to address these issues. The lack of standardized ethical frameworks and regulatory oversight hinders the safe and equitable integration of AI in clinical practice.

#### Rapidly evolving field

AI in health care is a rapidly evolving domain. Consequently, some studies may become outdated quickly, and newer developments might not be captured at the time of this review.

#### Language and database constraints

The review was limited to articles published in English and sourced from a select number of databases, potentially omitting relevant studies published in other languages or not indexed in the chosen repositories.

#### Use of retrospective datasets

Significant proportion of the studies included in this review are retrospective in nature. While retrospective analyses are valuable for generating hypotheses and offering early insights into the potential of artificial intelligence in cardiovascular diagnostics, they carry inherent limitations that affect real-world applicability. Retrospective studies often rely on curated, high-quality datasets obtained from single institutions or specialized research environments. These datasets typically feature fewer missing values, consistent image quality, and carefully selected patient populations. Although such conditions strengthen internal validity, they do not necessarily capture the variability, noise, and complexity of routine clinical practice.

As a result, AI models validated primarily on retrospective data may demonstrate impressive diagnostic accuracy under controlled conditions but risk underperforming in heterogeneous, real-world populations. Patient demographics, comorbidities, imaging artifacts, and workflow differences commonly encountered in clinical care can all influence model reliability. Moreover, retrospective designs are prone to selection bias and cannot capture prospective temporal relationships between diagnosis, intervention, and outcomes. This limits the ability to evaluate how AI integration impacts patient care pathways, clinical decision-making, and long-term outcomes.

The reliance on retrospective studies also hampers the assessment of safety and unintended consequences. Without prospective validation, it is difficult to determine whether AI models sustain accuracy across diverse populations or inadvertently reinforce systematic errors and inequities. For these reasons, retrospective evidence should be interpreted as preliminary. Future research must prioritize prospective, multicenter, real-world trials to confirm generalizability, reproducibility, and clinical utility. Until such studies are available, the translation of AI from promising innovation to reliable clinical practice remains incomplete.

### Lack of grey literature and clinical trials

Grey literature sources and clinical trial registries were not included. This decision was made deliberately to preserve the methodological rigor and reliability of the findings. Grey literature encompasses sources such as conference abstracts, dissertations, preprints, and non–peer-reviewed reports. While these can provide early insights, they often lack the detailed methodology, peer review, and quality assurance processes necessary for robust evidence synthesis. Similarly, although clinical trial registries such as ClinicalTrials.gov or the WHO International Clinical Trials Registry Platform may list ongoing or completed studies, the information available is frequently incomplete, lacking published results or peer-reviewed outcomes that could be critically appraised.

During the preliminary scoping phase, we sought to identify whether inclusion of such sources would meaningfully expand the evidence base. However, no unpublished or registry-listed trials met our predefined inclusion criteria of reporting diagnostic accuracy metrics or patient outcomes specific to AI in cardiovascular medicine. Incorporating incomplete data at this stage risked introducing bias, undermining transparency, and potentially overstating the efficacy of AI applications.

Excluding grey literature and registries also reduced the risk of duplication with later peer-reviewed publications of the same studies. Given the rapid pace of AI research, many preprints and abstracts are subsequently published in indexed journals with more comprehensive data. Thus, focusing on peer-reviewed studies ensured that the synthesis was based on the highest-quality, verifiable evidence available.

Nevertheless, this exclusion represents a limitation. Future systematic reviews may benefit from including grey literature and trial registries as the field matures and more unpublished studies provide robust, clinically relevant outcomes suitable for evaluation.

## Recent updates 2024–2025

### Uniqueness of this review compared to recent systematic reviews (updated 2024–2025)

While several systematic reviews on the role of AI in cardiovascular medicine have been published in 2024, our review distinguishes itself by offering more comprehensive/quantitative insights into AI’s application across multiple diagnostic modalities and emphasizing the ethical and practical challenges associated with its implementation. Below are key areas where our review provides additional insights and updated findings compared to other recent publications.

#### Quantitative results from selected studies

Unlike other 2024 reviews, which often discuss AI applications in general terms, our review focuses on presenting quantitative outcomes from the studies selected. The review goes beyond merely mentioning AI’s potential, by providing specific data on key metrics such as:

- Sensitivity and specificity of AI-assisted diagnostics
- PPVs and NPVs
- Diagnostic accuracy rates

Improvements in patient outcomes, such as reduced mortality and morbidity

For instance, studies included in our review, such as those by Knackstedt *et al*^[8]^ and Chen *et al*^[9]^, report concrete numerical improvements in diagnostic precision and patient outcomes, showcasing how AI surpasses traditional methods. This focus on quantitative evidence offers health care providers and researchers a more data-driven understanding of AI’s impact, making our review a valuable resource for clinical decision-making. Other reviews, while insightful, often lack this level of detail and fail to provide specific performance metrics across various AI applications.

#### Comprehensive focus on diagnostic modalities

Many recent reviews have focused on a specific aspect of AI, such as its application in echocardiography or ECG. For instance, Zargarzadeh *et al*^[1]^ provided a detailed review of AI’s role in echocardiography, while Romiti *et al*^[4]^ centered their review on ECG-based AI models. However, these reviews are often limited to one diagnostic tool, providing a narrower scope of evidence.

Our review goes beyond individual diagnostic tools by synthesizing evidence on the use of AI across multiple cardiovascular diagnostics, including

- Echocardiography
- ECG
- CT angiography
- Predictive analytics

This broader approach allows us to compare AI’s performance across different modalities and highlight how AI can complement traditional diagnostics in a variety of clinical settings. This integrative perspective makes our review a more comprehensive resource for clinicians looking to understand the overall potential of AI in cardiovascular care, rather than focusing on a single diagnostic method.

#### Updated evidence on predictive models and personalized medicine

A notable advancement in 2024 has been the rise of AI-based predictive analytics and personalized treatment approaches. Ahmad *et al*^[13]^ and Wallert *et al*^[12]^ previously explored AI’s role in prognostication, but our review incorporates more recent studies (e.g., Commandeur *et al*^[11]^) that use ML to predict long-term cardiovascular outcomes such as myocardial infarction and mortality. The inclusion of newer predictive models, including those using ML for risk stratification and tailored treatment decisions, highlights AI’s growing role in personalized medicine.

This focus on predictive modeling and its ability to forecast disease progression and treatment responses is less prominent in other reviews, positioning our paper as an updated and future-oriented review of AI’s capabilities in cardiovascular health care.

#### Detailed examination of ethical considerations and AI bias

While other 2024 reviews have touched on the technical aspects of AI, few have thoroughly discussed the ethical challenges that accompany AI implementation in cardiovascular diagnostics. Our review dedicates a significant section to issues such as:

- Algorithmic bias and its potential to perpetuate health care disparities
- Data privacy concerns, particularly given the large datasets required for AI models
- Transparency and the “black box” problem in AI decision-making, which affects both clinician trust and patient consent

In contrast to recent reviews (e.g., Lewin *et al*^[32]^), which briefly acknowledge ethical concerns, our review offers in-depth analysis supported by specific examples from the literature. This makes our paper an essential resource for health care providers, ethicists, and policymakers who are navigating the challenges of AI integration in clinical practice.

#### Discussion on real-world implementation challenges

Most 2024 reviews focus primarily on the theoretical benefits of AI, while our review goes further by addressing the practical challenges faced during real-world implementation. These challenges include:

1. Data variability and quality: Many studies use high-quality, curated datasets, which may not reflect the messiness of real-world clinical data.
2. Integration with clinical workflows: Our review highlights the barriers in incorporating AI tools into daily medical practice, including resistance from health care providers and issues with electronic health record (EHR) compatibility.
3. Regulatory hurdles: We also discuss the evolving regulatory landscape for AI, providing a more complete picture of what is needed for AI tools to transition from research to widespread clinical use.

This real-world focus is lacking in many of the 2024 reviews, which often limit their scope to AI’s theoretical benefits and diagnostic potential without addressing the implementation difficulties that health care providers face.

#### Inclusion of the most recent AI technologies and applications

Finally, our review incorporates evidence from the latest studies published in early 2024, providing up-to-date insights into the advancements in AI technologies. While previous reviews may cite foundational AI applications from 2020 to 2023, our inclusion of cutting-edge studies on DL-based echocardiography and CT angiography applications ensures that readers are informed of the most recent technological breakthroughs and their clinical implications.

By presenting a current and forward-looking view of AI in cardiovascular diagnostics, our review serves as an updated reference point for ongoing and future research, distinguishing it from other reviews that may already be outdated due to the fast pace of AI developments.

### Key recommendations for future research

#### Multi-center prospective validation

- Design large-scale, prospective, multi-center studies that recruit patients across different regions, health care systems, and demographic groups.
- Include both high-resource tertiary care centers and community/low-resource settings to test AI performance in varied environments.
- Mandate external validation datasets as part of study protocols, with subgroup analyses by age, sex, ethnicity, and comorbidity burden.

#### Explainability benchmarks

- Incorporate explainable AI (XAI) tools (e.g., saliency maps, SHAP values, feature importance plots) into study protocols.
- Report how clinicians interpret AI outputs, and whether explanations improve diagnostic confidence and trust.
- Develop standardized benchmarks (e.g., clinician–AI concordance scores) to evaluate interpretability across trials.

#### Standardized reporting guidelines

- Adhere to established extensions such as TRIPOD-AI, CONSORT-AI, and SPIRIT-AI in study design and reporting.
- Provide transparent details on dataset size, source, demographics, and inclusion/exclusion criteria.
- Consistently report diagnostic performance metrics, including sensitivity, specificity, AUC, PPV, NPV, and calibration curves.
- Encourage journals and funding agencies to enforce adherence to these frameworks as part of peer review and grant evaluation.

#### Integration of grey literature and registries

1. Systematically search and include preprints, conference abstracts, and trial registries to reduce publication bias.
2. Track registered trials (e.g., ClinicalTrials.gov, WHO ICTRP) and update systematic reviews as results become available.

#### Ethical and equity considerations

- Report subgroup analyses by sex, race/ethnicity, and socioeconomic status to identify and mitigate bias.
- Involve ethicists, patient representatives, and diverse clinicians early in study design to ensure fairness and transparency.
- Establish data-sharing agreements that allow independent auditing and re-validation of AI models for bias detection.

## Conclusion

This systematic review provides robust evidence supporting the beneficial impact of AI in enhancing diagnostic accuracy and patient outcomes in cardiovascular medicine. The findings highlight the revolutionary potential of AI, exploring the need for continued research and ethical considerations. ML and DL algorithms enhance diagnostic accuracy in cardiovascular medicine compared to traditional methods. AI models demonstrated higher sensitivity, specificity, and predictive values in various diagnostic modalities such as echocardiography, ECG, and cardiac imaging. Additionally, AI applications were associated with improved patient outcomes, including reduced morbidity and mortality, and more effective treatment planning.

## Data Availability

All data used in this systematic review are publicly available and sourced from previously published studies. No new data were generated for this work. All included articles have been appropriately cited within the manuscript and are available through the references section.

## Research registration unique identification number (UIN)

The research registration unique identification number (UIN) was not required because it is a systematic review.
